# Prognostic value of computed tomography-derived fractional flow reserve in patients with diabetes mellitus and unstable angina

**DOI:** 10.1186/s12933-024-02493-8

**Published:** 2024-11-08

**Authors:** Qi Zhao, Li Liu, Huimin Xian, Xing Luo, Donghui Zhang, Shenglong Hou, Chao Qu, Ruoxi Zhang, Xiufen Qu

**Affiliations:** 1 Department of Cardiology, The First Affiliated Hospital of Harbin Medical University, Harbin, 150086 China; 2 Department of Cardiology, The Second Affiliated Hospital of Harbin Medical University, Harbin, 150086 China; 3 Department of Cardiology, Heilongjiang Province Hospital, Harbin, 150036 China; 4 Department of Cardiology, Harbin Yinghua Hospital, Harbin, 150086 China

**Keywords:** Computed tomography-derived fractional flow reserve, Diabetes mellitus, Unstable angina, Calcific lesion, Clinical prognosis

## Abstract

**Background:**

Coronary artery calcification is commonly found in patients with type 2 diabetes mellitus (T2DM), which may compromise the diagnostic accuracy of coronary computed tomography angiography (CTA). Computed tomography-derived fractional flow reserve (CT-FFR), which integrates coronary anatomy with functional assessment, holds the potential to become a powerful diagnostic tool for evaluating calcified lesions.

**Objective:**

We aim to assess the prognostic value of CT-FFR for calcific lesions in patients with T2DM and unstable angina (UA).

**Methods:**

We conducted a retrospective study involving 3,392 patients who were diagnosed with T2DM and UA who underwent coronary CTA, with at least one visible calcification site. Of those, 1,091 patients and 1,372 vessels were recommended by cardiovascular specialists and completed invasive coronary angiography (ICA) and invasive fractional flow reserve (FFR) measurements. Simultaneously, those patients also underwent CT-FFR measurements and were divided into two groups based on CT-FFR values: one group with CT-FFR > 0.80 and the other with CT-FFR ≤ 0.80. Demographics, clinical data, the diagnostic performance of CT-FFR, analysis of calcified lesions on CTA, and major adverse events during follow-up were recorded.

**Results:**

The diagnostic accuracy, sensitivity, specificity, positive predictive value (PPV), negative predictive value (NPV), and the area under the curve (AUC) of CT-FFR were 84.8%, 84.6%, 85.1%, 84.7%, 85.0%, and 84.8%, respectively, per patient, and 82.2%, 80.3.2%, 81.8%, 79.7%, 81.1%, and 82.9% respectively, per vessel. For lesion and calcification characteristics, the degree of stenosis, lesion length, rate of bifurcation lesions, diffusive lesions, occlusion, calcium volume, and coronary artery calcification score (CACS) were significantly higher in the CT-FFR ≤ 0.8 group compared to the CT-FFR > 0.8 group. In contrast, the minimum cross-sectional area was smaller in the CT-FFR ≤ 0.8 group than in the CT-FFR > 0.8 group. Major adverse cardiovascular and cerebrovascular events (MACCE) at the 3-year follow-up was significantly higher in the CT-FFR ≤ 0.8 group compared to the CT-FFR > 0.8 group. The CT-FFR value is an independent predictor of MACCE at the 3-year follow-up.

**Conclusion:**

CT-FFR demonstrated significant diagnostic performance using invasive FFR as the reference standard and proved to be an important predictive tool for assessing prognosis not only in calcified lesions but also in lesions with a CACS score of zero in patients with T2DM and UA. CT-FFR may serve as a valuable tool for guiding treatment decisions in these patients.

**Graphical abstract:**

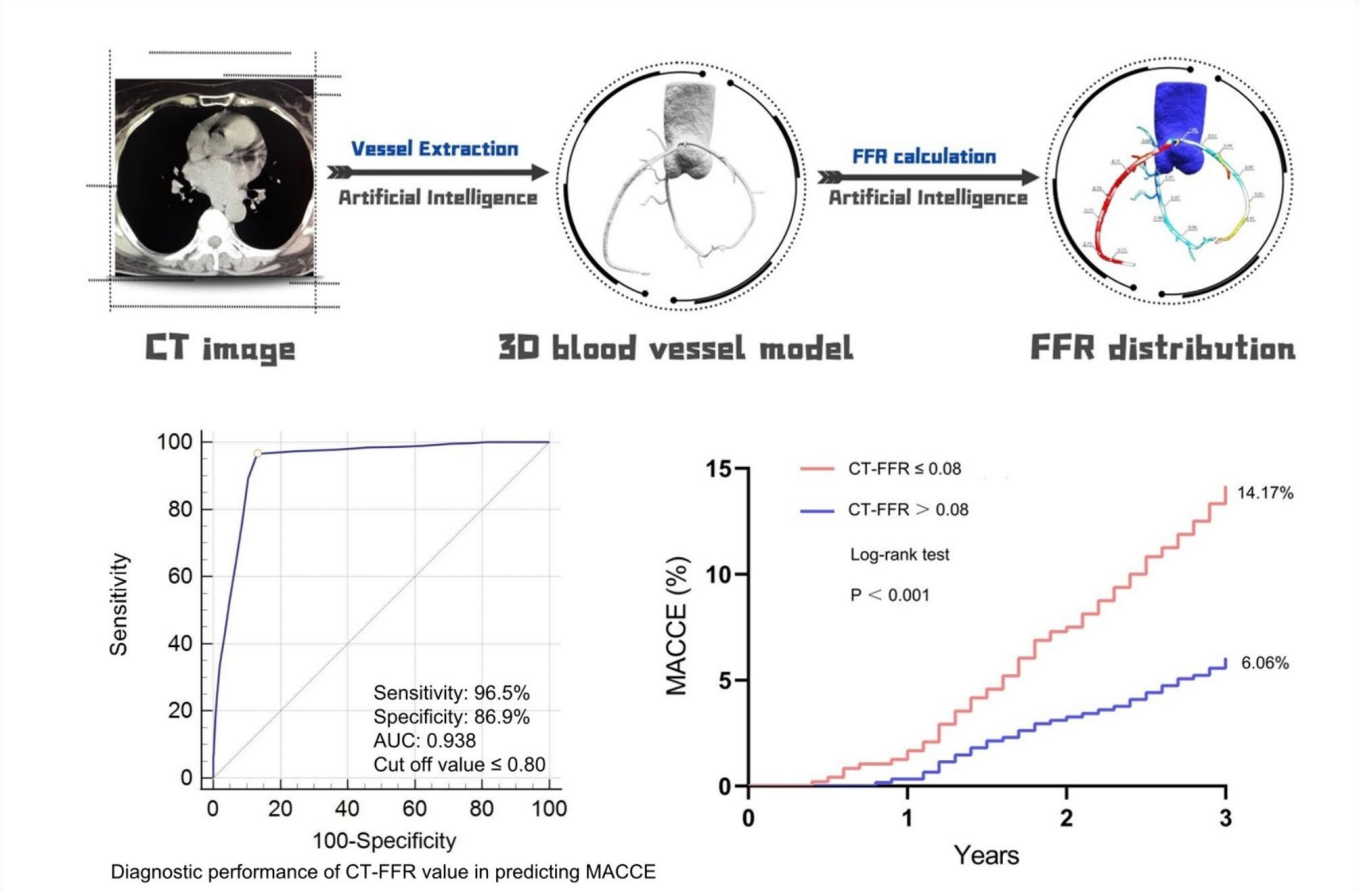

## Introduction

Type 2 diabetes mellitus (T2DM) is linked to a two- to fourfold increased risk of coronary artery disease (CAD) compared to nondiabetic populations and is considered an independent risk factor for the condition [1]. Blood vessels in patients with T2DM exhibited increased deposition of connective tissue and calcium-phosphate salts in the atherosclerotic intima and media, resulting in the calcific lesion [2, 3]. Coronary calcific lesions serve as an indicator of coronary atherosclerosis and can be non-invasively evaluated using ECG-gated non-contrast CT of the heart [4]. There is a positive correlation between the presence and extent of calcific lesions and the prevalence of CAD as determined by coronary computed tomography angiography (CTA) [5], as well as the risk of future cardiovascular events [6, 7]. However, in the assessment of coronary arteries, a high burden of the calcific lesion can diminish the diagnostic performance of coronary CTA in terms of vascular anatomy, particularly by reducing its specificity and positive predictive value. Consequently, it may be less effective in identifying patients without significant coronary artery stenosis [4, 8].

Computed tomography-derived fractional flow reserve (CT-FFR) is a noninvasive functional test providing anatomical and functional evaluation of the overall coronary tree. Numerous clinical trials have shown that the diagnostic accuracy of coronary CTA improves with the addition of CT-FFR, largely due to its superior specificity compared to coronary CTA alone [9–11]. This advancement could reduce the necessity for invasive coronary angiography (ICA) in patients presenting with chest pain [12]. Additionally, some studies suggest that incorporating CT-FFR into decision-making is safe, with an CT-FFR value of ≥ 0.8 linked to favorable outcomes [13, 14]. The development and integration of CT-FFR have allowed coronary CTA to evaluate both anatomical and physiological aspects, akin to ICA and invasive FFR [15]. Therefore, we retrospectively analyzed CT-FFR for calcified lesions in patients with T2DM and unstable angina (UA), and evaluated clinical outcomes at 3 years.

## Methods

### Ethics statement

The study protocol conformed to the ethical guidelines of the 1975 Declaration of Helsinki and its later amendments. This retrospective study was conducted with the approval of the Research Ethics Committee of the First Affiliated Hospital of Harbin Medical University, the Second Affiliated Hospital of Harbin Medical University, and Heilongjiang Provincial People's Hospital, China (Approval number is SYDWGZR-2020–128). The retrieved data originated from the imaging systems of each hospital for those who had undergone coronary CTA scans for analysis. The follow-up information of the patients was obtained through outpatient visits or telephone follow-ups. Each patient provided written informed consent for undergoing the procedures and for having their data collected and analyzed for research purposes.

### Study design and participants

**Fig. 1:**
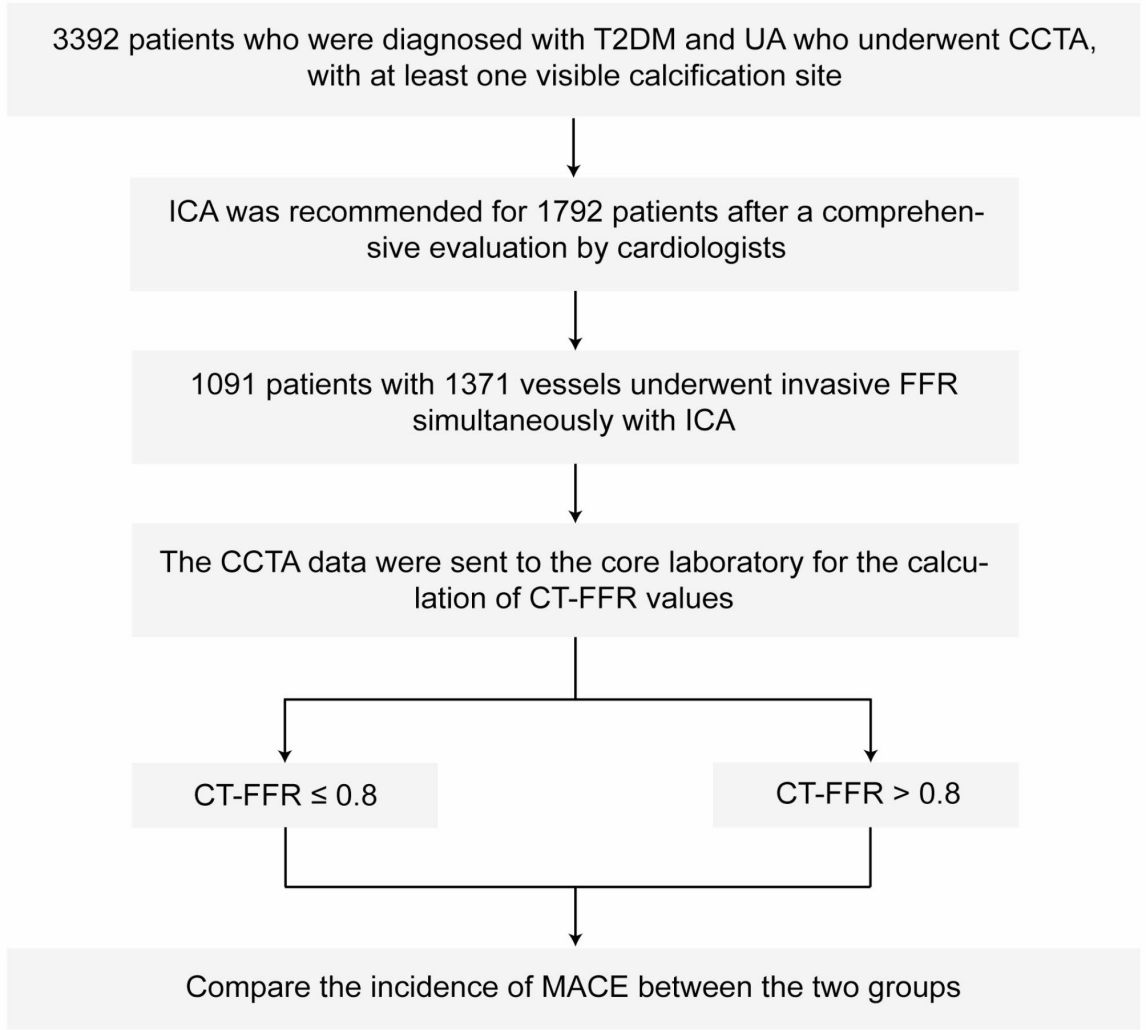
Study flowchart. T2DM, type 2 diabetes mellitus; UA, unstable angina; CCTA, coronary computed tomography angiography; ICA, invasive coronary angiography; CT-FFR, computed tomography-derived computed fractional flow reserve; MACCE, major adverse cardiovascular and cerebrovascular events

Between January 2016 and December 2020, we conducted a retrospective study involving 3,392 T2DM patients with symptoms of angina who underwent coronary CTA and were diagnosed with UA, with at least one visible calcification site detected via CTA imaging, which includes both microcalcifications and localized calcifications identified by imaging specialists. Of these, 1,792 patients were recommended to undergo ICA based on a comprehensive assessment by cardiovascular specialists. Ultimately, 1,091 patients and 1,372 vessels underwent ICA, with angiography being accompanied by invasive FFR measurements (Fig. 1). The exclusion included acute coronary syndrome, chronic congestive heart failure, left main coronary artery disease, severe liver and kidney dysfunction, and patients with a life expectancy < 3 years.

Patients were divided into two groups based on their CT-FFR values: those with CT-FFR > 0.80 and those with CT-FFR ≤ 0.80. The CT-FFR values for the three main coronary arteries (left anterior descending artery, left circumflex artery, and right coronary artery) and their branches were measured for each patient. The lowest value obtained was recorded and used as the test value for analysis.

### Coronary CTA scanning protocols

Each participating medical center will perform coronary CTA using 256-detector row CT scanners (GE Revolution, General Electric Medical Systems, USA). The scanning procedures will adhere to each center’s established coronary CTA clinical practices. Standard administration of nitroglycerin and beta-blockers will be according to each center’s protocol. After the coronary CTA, an electronic questionnaire will collect baseline details for each patient, including the type of scanning equipment, protocols, iodinated contrast injection details (name, concentration, dosage, and rate), average heart rate during scanning, and the use of nitroglycerin and beta-blockers. The Digital Imaging and Communications in Medicine (DICOM) files will be transmitted to the core laboratory, either online or offline, for preliminary image quality and coronary stenosis evaluation. Following a thorough review, patients meeting the inclusion criteria will be officially enrolled and notified by the core laboratory [16].

Prior to the coronary CTA, a coronary calcium scoring study without contrast enhancement will be conducted. Measurements and calculations were performed using the GE Revolution CT scanner and its SmartScore software. Coronary artery coronary artery calcification score (CACS) analysis was conducted under the AJ130 threshold model, applying the Agatston scoring method to calculate the CACS values [17].

### Scan parameters, contrast injection protocols, and reconstruction parameters

Calcium Scoring Scan Parameters:

1. Scanner: A 64-slice multidetector CT scanner was used.
2. Tube Voltage: 120 kVp
3. Tube Current: 200 mAs
4. Slice Thickness: 2.5 mm
5. Reconstruction Algorithm: A standard filtered back-projection algorithm was applied to assess the coronary artery calcification.

CT Angiography Scan Parameters:

1. Tube Voltage: 100–120 kVp (adjusted based on patient body mass index)
2. Tube Current: 600–700 mAs
3. Slice Thickness: 0.625 mm
4. Rotation Time: 0.35 s
5. Pitch: 0.2–0.3
6. Reconstruction Algorithm: Images were reconstructed using iterative reconstruction techniques to enhance image quality, particularly in the presence of high-density calcifications.

Contrast Injection Protocol:

1. Contrast Agent: Iodinated contrast medium (350–370 mg I/mL)
2. Injection Rate: 4–5 mL/second, followed by a 30–50 mL saline flush at the same rate.
3. Volume: Total contrast volume was typically between 50–70 mL, depending on patient body weight and scan length.
4. Timing: Bolus tracking was used to optimize the timing of the contrast-enhanced scan. A region of interest (ROI) was placed on the ascending aorta, and scanning commenced when the contrast reached a predefined threshold.

Heart Rate Control:

1. Beta-Blockers: Administered as needed to reduce heart rate below 65 beats per minute prior to the scan.
2. Nitroglycerin: Sublingual nitroglycerin (0.4 mg) was administered 2–3 min prior to scanning to achieve coronary vasodilation.

Reconstruction Parameters:

1. Kernel: A medium-smooth kernel (B46f) was used for calcium scoring, while a sharper kernel (B26f) was applied for coronary CTA.
2. Slice Thickness: 0.6 mm with a 0.4 mm increment for coronary CTA reconstruction to ensure high spatial resolution.
3. Reconstruction Field of View (FOV): Limited to the heart to reduce noise and improve resolution.

### CT-FFR measurements

**Fig. 2:**
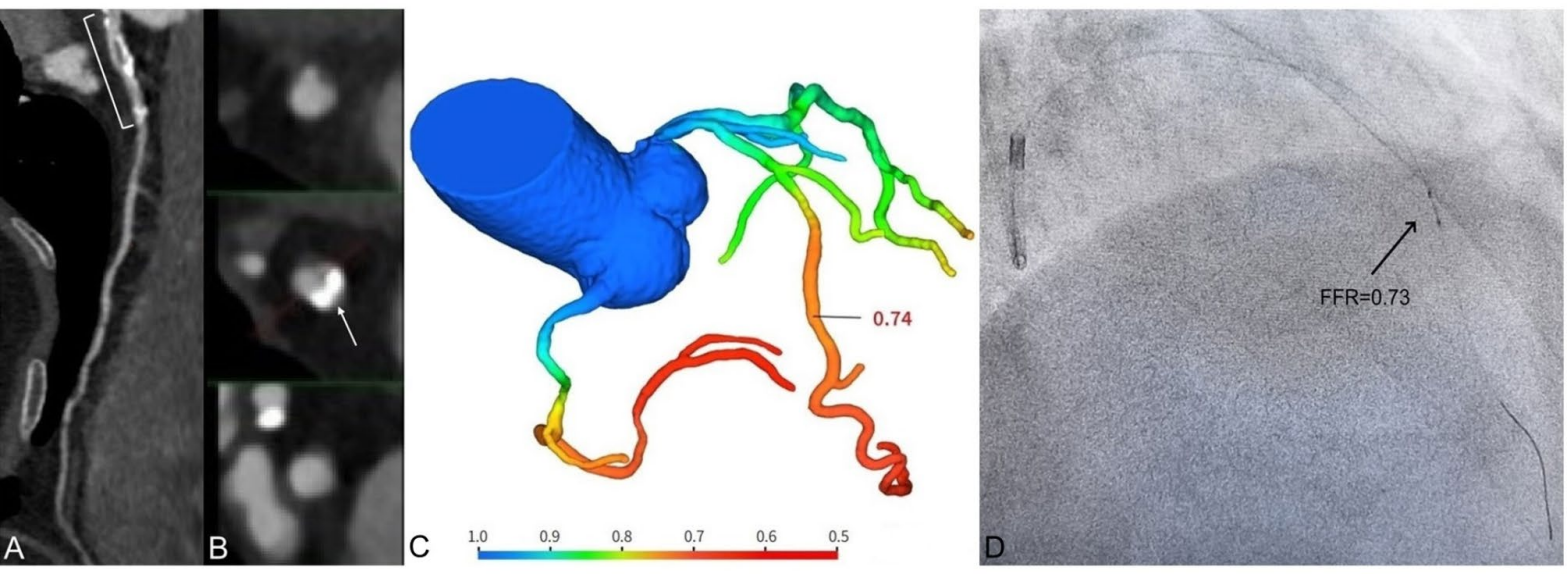
A 72-year-old patient underwent CTA, CT-FFR, and FFR examinations. (**A**) The axial and curved planar reformatted images of the calcified segment for LAD (the bracket); (**B**) The cross-sectional and curved planar reformatted images of the calcified segment for LAD (the arrow); (**C**) The CT-FFR value of the mid-segment of the LAD was 0.74; (**D**) The FFR value of the mid-segment of the LAD was 0.73. CTA, coronary computed tomography angiography; CT-FFR, computed tomography-derived computed fractional flow reserve; LAD, left anterior descending artery

All CT-FFR values will be calculated using automated software (DeepVessel-FFR from Keya Medical Technology Co., Ltd.). This software includes two primary components: the coronary artery segmentation model and the computational fluid dynamics (CFD) simulation model (Fig. 2A–C). Specifically, a modified V-Net is initially employed to segment the coronary arteries from the coronary CTA images [18]. First, using established anatomical guidelines and prior vessel segmentation data, we label the vascular branches. A simplified CFD model is then applied to calculate blood flow and pressure, automatically generating CT-FFR values along the coronary arteries. CT-FFR assessments for coronary arteries with diameters of ≥ 1.8 mm will be conducted in the core laboratory by two cardiovascular radiologists. An additional experienced observer will confirm the identification of coronary plaques and the extent of lumen stenosis. CT-FFR measurements will be taken at the proximal and distal points of the stenosis, as well as 20 mm beyond the stenosis to the end of the target vessel (with a minimum diameter of 1.8 mm). A CT-FFR ≤ 0.80 indicates hemodynamically significant coronary stenosis. In this study, lesion-specific CT-FFR values were used for analysis. Specifically, patients with a CT-FFR ≤ 0.80, measured 20 mm distal to the stenosis, were considered to have lesion-specific ischemia. On the other hand, those with a CT-FFR ≤ 0.80 at the end of the target vessel were classified as having distal vessel ischemia. However, for the purpose of analysis, we focused on the lesion-specific CT-FFR values.

### Invasive coronary angiography

All ICAs were performed at the following hospitals using a standard protocol [19]: the First Affiliated Hospital of Harbin Medical University, the Second Affiliated Hospital of Harbin Medical University, and Heilongjiang Provincial People's Hospital, all of which are tertiary interventional centers conducting over 5,000 ICAs annually. The procedures were carried out by experienced interventional cardiologists, each performing more than 1,000 ICAs per year, who were not involved in this study. Data on baseline demographics, angiographic characteristics, and laboratory and physical examination outcomes during hospitalization were systematically reviewed from the medical records maintained by each hospital.

### Invasive FFR

All major coronary arteries were routinely assessed using FFR, regardless of stenosis severity, except in cases of occluded or subtotal lesions exceeding 90%. Intracoronary adenosine (150 mg) or intravenous adenosine infusion (140 ug/kg/min) was administered to induce maximal coronary hyperemia [20]. FFR was calculated as the ratio of mean distal intracoronary pressure to mean arterial pressure (Fig. 2D). A coronary lesion was deemed hemodynamically significant if the FFR was ≤ 0.80 or if stenosis severity exceeded 90% as determined by quantitative coronary angiography when FFR was unavailable. Lesions with an FFR > 0.80 or a stenosis severity < 30% (as measured by quantitative coronary angiography) in the absence of FFR measurements were considered not functionally significant. All images and FFR signals were interpreted by experienced interventional cardiologists who were blinded to the imaging results [16].

### Definition

UA is defined as acute angina while at rest (within the 48 h before presentation), subacute angina while at rest (within the previous month but not within the 48 h before presentation), or new onset of accelerated (progressively more severe) angina; the clinical circumstances in which unstable angina develops, defined as either angina in the presence or absence of other conditions (e.g., anemia, fever, hypoxia, tach ycardia, or thyrotoxicosis) or angina within two weeks after an acute myocardial infarction; and whether or not electrocardiographic abnormalities are present [21]. DM was diagnosed if a patient was taking insulin or oral hypoglycemic drugs, or if not on these medications, had a casual plasma glucose level > 11.1 mmol/L, a fasting plasma glucose level > 7 mmol/L, or a glycosylated hemoglobin level > 6.0%. Hypertension was diagnosed if the systolic blood pressure was ≥ 140 mmHg and/or diastolic blood pressure was ≥ 90 mmHg, or if the patient had been on antihypertensive medication long-term. Hyperlipidemia was defined as a fasting total serum cholesterol level > 5.17 mmol/L, a low-density lipoprotein cholesterol > 3.15 mmol/L, or a serum triglyceride level > 1.70 mmol/L, or if the patient was on lipid-lowering medication due to a history of hypercholesterolemia. Smoking status was defined as current regular use of cigarettes or having quit smoking within the past year [22]. Repeat revascularization is defined as any subsequent revascularization procedure (such as PCI] or coronary artery bypass grafting [CABG]) performed after the initial revascularization [23]. Major adverse cardiovascular and cerebrovascular events (MACCE) were defined to include all-cause mortality, cardiac death, non-fatal myocardial infarction (MI), stroke, and repeat revascularization.

### Statistical analysis

Quantitative data are presented as mean ± standard deviation, while qualitative data are shown as frequency (percentage). Comparisons between groups were performed using the independent two-sample *t*-test. The chi-square test or Fisher’s exact test, as appropriate, was used for categorical variables. The Pearson correlation coefficient was used to determine the correlation between CT-FFR and invasive FFR. A linear regression was used to analyze the correlation between CT-FFR values and CACS. For the regression analysis, we categorized CT-FFR values into two groups: CT-FFR ≤ 0.8 and CT-FFR > 0.8. This binary classification was used in both univariate and multivariate logistic regression analyses to identify predictors of MACCE. The threshold of 0.8 is commonly used to signify hemodynamically significant coronary lesions, allowing for a clear comparison of outcomes between the two groups. A receiver operating characteristic (ROC) curve was generated to determine the optimal cutoffs for indicators with the best diagnostic sensitivity and specificity. The Kaplan–Meier (K-M) method was used to estimate follow-up outcomes, and the log-rank test assessed differences in MACCE distributions. Two-sided *P*-values < 0.05 were considered statistically significant. All statistical analyses were conducted using SPSS version 22.0 (SPSS Inc., Chicago, IL, USA).

## Results

### Baseline characteristics

The screening process involved 3,392 patients, among whom 1,091 were finally enrolled in the study between January 2016 and December 2020. A comparison of the two groups indicated a difference regarding sex, as patients had a lower rate of female in the CT-FFR ≤ 0.8 group (*P* = 0.028). There was no significant difference in other characteristics of the two group’s baseline demographics (Table 1).

**Table 1:** Patient demographics and clinical data

	CT-FFR ≤ 0.8 (n = 480)	CT-FFR > 0.8 (n = 611)	*P*-values
Age (years)	63.8 ± 5.8	64.1 ± 6.0	0.387
Female, n (%)	215 (44.79)	315 (51.55)	0.028
BMI (kg/m^2^)	25.8 ± 3.1	25.9 ± 2.9	0.603
Cardiovascular risk factors, n (%)			
Hypertension	308 (64.17)	391 (63.99)	1.000
Hyperlipidemia	310 (64.58)	370 (60.56)	0.186
Current smoking	283 (58.96)	361 (59.08)	1.000
Clinical data			
SBP (mmHg)	119.9 ± 19.4	120.5 ± 19.2	0.603
DBP (mmHg)	72.9 ± 13.6	72.7 ± 13.6	0.796
Total cholesterol (mol/L)	4.51 ± 0.81	4.50 ± 0.80	0.896
LDL-cholesterol (mol/L)	3.02 ± 1.14	3.01 ± 1.10	0.917
HDL-cholesterol (mol/L)	1.31 ± 0.50	1.31 ± 0.49	0.761
Triglyceride (mol/L)	2.21 ± 0.96	2.22 ± 0.97	0.807
HbA1c (%)	6.5 ± 1.70	6.4 ± 1.59	0.743
hs-CRP	6.20 ± 1.49	6.26 ± 1.43	0.495
NT-proBNP (pg/mL)	103.2 ± 17.9	97.9 ± 13.1	0.252

Mean values ± standard deviation, median (interquartile range), and % (n) were reported for variables, respectively. BMI, body mass index; SBP, systolic blood pressure; DBP, diastolic blood pressure; WBC, white blood cell; HDL, high-density lipoprotein; HbA1c, glycosylated hemoglobin; hs-CRP, high-sensitivity C-reactive protein; LDL, low-density lipoprotein; NT-proBNP, N-terminal pro-brain natriuretic peptide

### Diagnostic performance for CT-FFR

The lesion specific ischemia diagnosis for CT-FFR compared with CTA diameter stenosis was shown in Table 2, with FFR as reference standard. At the patient level, the diagnostic accuracy, sensitivity, specificity, PPV, NPV, and AUC of CT-FFR were 84.8%, 84.6%, 85.1%, 84.7%, 85.0%, and 84.8% respectively, for CT-FFR, and 76.2%, 70.5%, 65.8%, 68.2%, 72.3%, and 73.1% respectively, for CTA. At the vessel level, the diagnostic accuracy, sensitivity, specificity, PPV, NPV, and AUC of CT-FFR were 82.2%, 80.3%, 81.8%, 79.7%, 81.1%, and 82.9% respectively, for CT-FFR, and 72.5%, 69.8%, 65.0%, 67.2%, 70.1%, and 71.1% respectively, for CTA.

**Table 2:** The lesion specific ischemia diagnosis for CT-FFR compared with CTA diameter stenosis

	Per-patient			Per-vessel		
	CTA	CT-FFR	*P*-value	CTA	CT-FFR	*P*-value
Accuracy	0.762 (0.710–0.814)	0.848 (0.819–0.874)	0.012	0.725 (0.680–0.770)	0.822 (0.790–0.854)	0.014
Sensitivity	0.705 (0.668–0.742)	0.846 (0.810–0.878)	< 0.001	0.698 (0.655–0.740)	0.803 (0.770–0.836)	< 0.001
Specificity	0.658 (0.621–0.695)	0.851 (0.813–0.884)	< 0.001	0.650 (0.615–0.685)	0.818 (0.780–0.856)	< 0.001
PPV	0.682 (0.642–0.722)	0.847 (0.811–0.878)	< 0.001	0.672 (0.630–0.710)	0.797 (0.760–0.834)	< 0.001
NPV	0.723 (0.684–0.762)	0.850 (0.812–0.883)	< 0.001	0.701 (0.660–0.740)	0.811 (0.780–0.842)	< 0.001
AUC	0.731 (0.694–0.768)	0.848 (0.817–0.903)	0.007	0.711 (0.660–0.762)	0.829 (0.790–0.868)	0.006

CT-FFR, computed tomography-derived fractional flow reserve; CTA, computed tomography angiography; PPV, positive predicted value; NPV, negative predicted value; AUC, the area under the curve

**Fig. 3:**
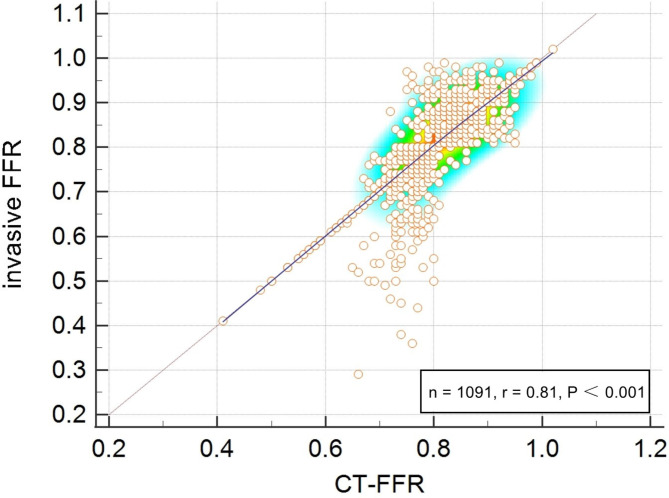
Scatter plot of invasive FFR and CT-FFR at patient level. CT-FFR, computed tomography-derived computed fractional flow reserve

The diagnostic performance for CT-FFR across CACS categories was shown in Table 3, with invasive FFR as reference standard. The diagnostic accuracy, sensitivity, specificity, positive predictive value (PPV), negative predictive value (NPV), and the area under the curve (AUC) of CT-FFR were 84.8%, 84.6%, 85.1%, 84.7%, 85.0%, and 84.8% respectively, per patient, and 82.2%, 80.3%, 81.8%, 79.7%, 81.1%, and 82.9% respectively, per vessel. The diagnostic performance of CT-FFR across different CACS categories (0, > 0 to < 100, ≥ 100 to < 400, and ≥ 400) showed no statistically significant differences at both the per-patient and per-vessel levels. The CT-FFR values were correlated well with values from invasive FFR (Pearson correlation coefficient r = 0.81, *P* < 0.001) (Fig. 3).

**Table 3:** Diagnostic performance and accuracy of CT-FFR across CACS categories on per-patient and per-vessel level

	n% (N/M)	CACS	Accuracy	Sensitivity	Specificity	PPV	NPV	AUC
Per-patient								
Total	100% (1091/1091)	60 (0, 645)	0.848 (0.819–0.874)	0.846 (0.810–0.878)	0.851 (0.813–0.884)	0.847 (0.811–0.878)	0.850 (0.812–0.883)	0.848 (0.817–0.903)
0	40.1% (437/1091)	0	0.854 (0.820–0.889)	0.832 (0.803–0.861)	0.845 (0.808–0.880)	0.859 (0.822–0.895)	0.838 (0.802–0.873)	0.846 (0.812–0.880)
> 0 to < 100	13.1% (143/1091)	30 (11, 60)	0.840 (0.810–0.870)	0.855 (0.818–0.892)	0.828 (0.800–0.856)	0.842 (0.805–0.879)	0.853 (0.812–0.894)	0.837 (0.805–0.868)
≥ 100 to < 400	15.4% (168/1091)	233 (159, 294)	0.850 (0.815–0.885)	0.832 (0.804–0.860)	0.844 (0.810–0.878)	0.851 (0.812–0.890)	0.836 (0.805–0.867)	0.860 (0.822–0.898)
≥ 400	31.4% (343/1091)	1219 (712, 2110)	0.848 (0.814–0.882)	0.834 (0.805–0.863)	0.856 (0.820–0.892)	0.841 (0.808–0.874)	0.849 (0.816–0.882)	0.837 (0.804–0.870)
*P*-value	-	-	0.376	0.589	0.163	0.492	0.371	0.258
Per-vessel								
Total	100% (1372/1372)	60 (0, 698)	0.822 (0.790–0.854)	0.803 (0.770–0.836)	0.818 (0.780–0.856)	0.797 (0.760–0.834)	0.811 (0.780–0.842)	0.829 (0.790–0.868)
0	40.8% (560/1372)	0	0.812 (0.780–0.844)	0.825 (0.790–0.860)	0.805 (0.770–0.840)	0.817 (0.780–0.854)	0.788 (0.760–0.816)	0.820 (0.780–0.840)
> 0 to < 100	12.2% (167/1372)	30 (14, 60)	0.822 (0.780–0.864)	0.808 (0.770–0.846)	0.804 (0.760–0.828)	0.816 (0.780–0.852)	0.830 (0.790–0.870)	0.801 (0.770–0.832)
≥ 100 to < 400	14.8% (203/1372)	225 (156, 294)	0.810 (0.780–0.840)	0.827 (0.790–0.864)	0.800 (0.770–0.830)	0.819 (0.780–0.858)	0.805 (0.760–0.830)	0.832 (0.790–0.874)
≥ 400	32.2% (442/1372)	1319 (724.5, 2533)	0.811 (0.780–0.842)	0.823 (0.790–0.856)	0.798 (0.760–0.836)	0.826 (0.780–0.872)	0.812 (0.770–0.854)	0.804 (0.770–0.838)
*P*-value	-	-	0.496	0.167	0.374	0.582	0.375	0.253

N, numerator; D, denominator; CT-FFR, computed tomography-derived fractional flow reserve; CACS, coronary artery calcification score; PPV, positive predicted value; NPV, negative predicted value; AUC, the area under the curve

### Comparison of lesion and calcification characteristics

**Fig. 4:**
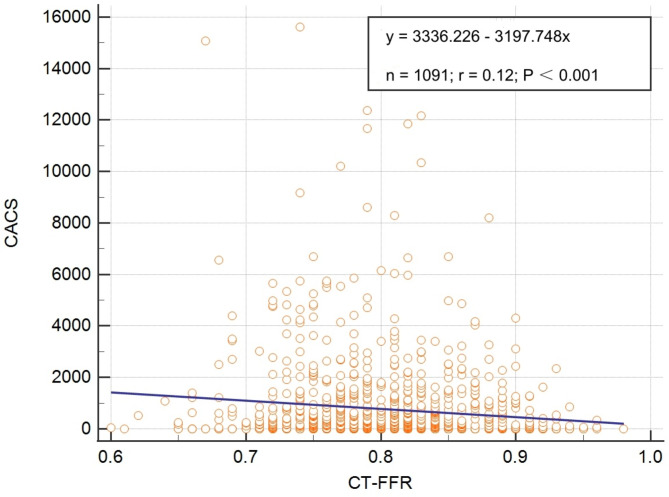
The linear regression analysis of CT-FFR value and CACS. CT-FFR, computed tomography-derived computed fractional flow reserve; CACS, coronary artery calcification score

For lesion and calcification characteristics, the degree of stenosis, lesion length, rate of bifurcation lesions, diffusive lesions, occlusion, calcium volume, and CACS were significantly higher in the CT-FFR ≤ 0.8 group compared to the CT-FFR > 0.8 group. In contrast, the minimum cross-sectional area was smaller in the CT-FFR ≤ 0.8 group than in the CT-FFR > 0.8 group (Table 4). A linear regression analysis showed a weak correlation between CT-FFR values and CACS, with an r-value of 0.12 and a statistically significant *P*-value of < 0.001 (Fig. 4).

**Table 4:** Comparison of lesion and calcification characteristics

	CT-FFR ≤ 0.8 (n = 480)	CT-FFR > 0.8 (n = 611)	*P*-values
Lesion characteristics			
Degree of Stenosis (%)	59.24 ± 18.35	45.59 ± 17.07	< 0.001
Minimum cross-sectional area (mm^2^)	3.64 (2.49, 5.04)	5.16 (3.76, 6.84)	< 0.001
Lesion Length (mm)	19.50 (10.80, 33.37)	15.13 (9.06, 26.44)	< 0.001
Bifurcation lesion, n (%)	247 (51.5)	129 (21.1)	< 0.001
Diffusive lesion, n (%)	150 (31.3)	119 (19.5)	< 0.001
Occlusion, n (%)	8 (1.7)	2 (0.3)	0.026
Calcification characteristics			
Calcium volume (mm^3^)	3.46 (0.00, 23.83)	0.45 (0.00, 12.29)	< 0.001
CACS (AJ130)	147.5 (0.0, 993.5)	19 (0.0, 530.0)	< 0.001

Mean ± standard deviation, median (interquartile range), and % (n) were reported for variables, respectively. CACS, coronary artery calcium score

### Clinical outcomes in 3-year follow-up

**Fig. 5:**
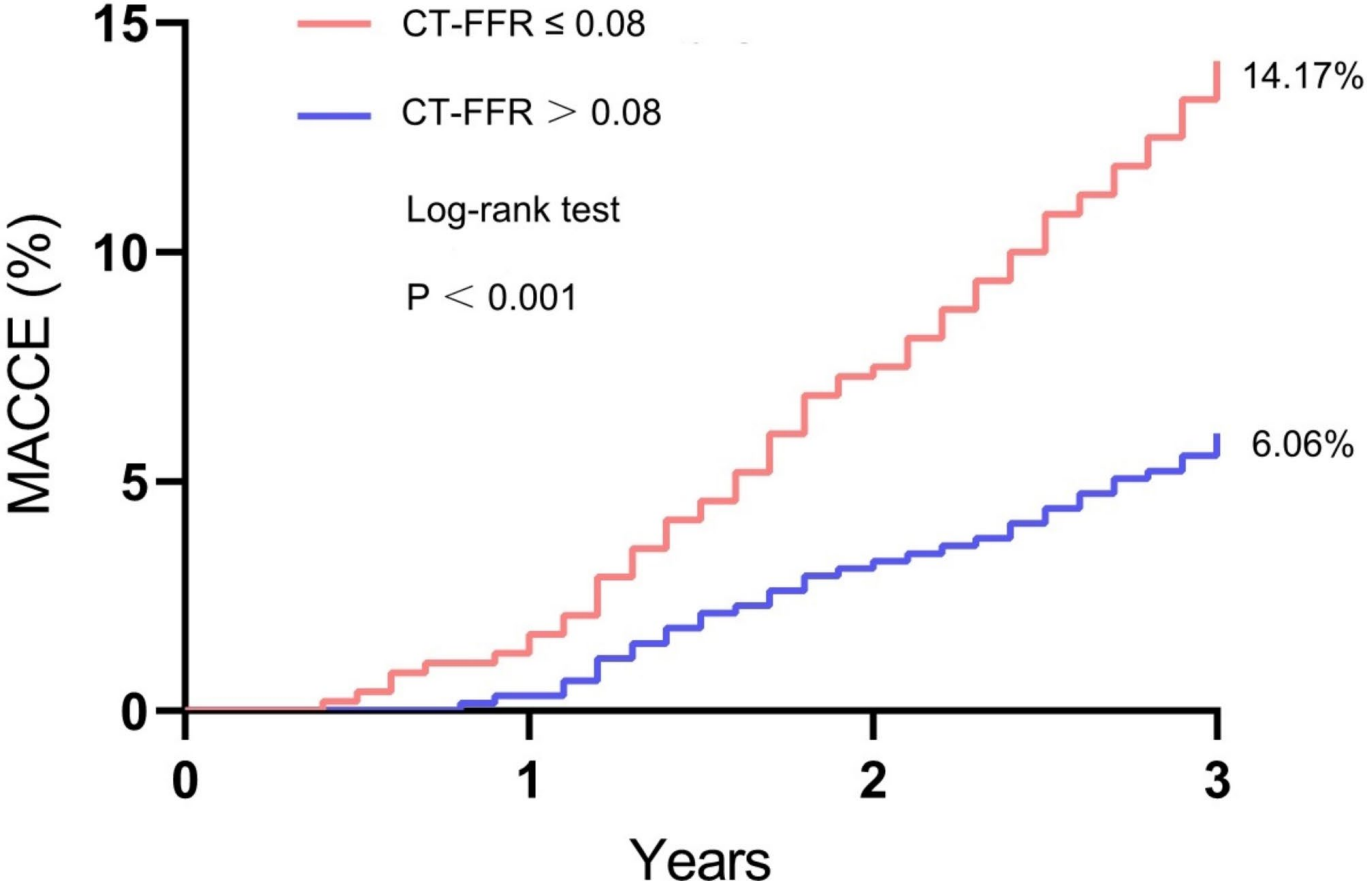
Kaplan–Meier curves for all-cause MACCE. MACCE, major adverse cardiovascular and cerebrovascular events

MACCE at the 3-year follow-up was significantly higher in the CT-FFR ≤ 0.8 group compared to the CT-FFR > 0.8 group (Table 5 and Fig. 5). Table 6 shows that there is no statistically significant difference in 3-year MACCE outcomes between patients with both CT-FFR and invasive FFR ≤ 0.8. CT-FFR value (Odds Ratio, 0.372; 95% confidence interval [0.233–0.591]; *P* < 0.001) was independently related with MACCE (Table 7).

**Table 5:** MACCE in 3-year follow-up

n, (%)	CT-FFR ≤ 0.8 (n = 480)	CT-FFR > 0.8 (n = 611)	*P*-values
MACCE	68 (14.17)	37 (6.06)	< 0.001
All-cause mortality	10 (2.08)	8 (1.31)	0.346
Cardiac death	5 (1.04)	2 (0.33)	0.251
Non-fatal MI	11 (2.29)	5 (0.82)	0.072
Stroke	5 (1.04)	5 (0.82)	0.756
Repeat revascularization	37 (7.71)	17 (2.78)	< 0.001
PCI	33 (6.88)	15 (2.45)	0.001
CABG	4 (0.83)	2 (0.33)	0.414

Mean values ± standard deviation, median (interquartile range), and % (n) were reported for variables, respectively. MACCE, major adverse cardiac and cerebrovascular events; MI, myocardial infarction, PCI, percutaneous coronary intervention; CABG, coronary artery bypass grafting

**Table 6:** 3-year MACCE outcomes in patients with CT-FFR and invasive FFR ≤ 0.8

n, (%)	CT-FFR ≤ 0.8 (n = 480)	invasive FFR ≤ 0.8 (n = 457)	*P*-values
MACCE	68 (14.17)	62 (13.57)	0.850
All-cause mortality	10 (2.08)	10 (2.19)	1.000
Cardiac death	5 (1.04)	5 (1.09)	1.000
Non-fatal MI	11 (2.29)	11 (2.41)	1.000
Stroke	5 (1.04)	4 (0.88)	1.000
Repeat revascularization	37 (7.71)	32 (7.00)	0.709
PCI	33 (6.88)	29 (6.35)	0.793
CABG	4 (0.83)	3 (0.66)	1.000

Mean values ± standard deviation, median (interquartile range), and % (n) were reported for variables, respectively. MACCE, major adverse cardiac and cerebrovascular events; MI, myocardial infarction, PCI, percutaneous coronary intervention; CABG, coronary artery bypass grafting

**Table 7:** Univariate and multivariate regression analysis for MACCE in 3-year follow-up

	Univariate		Multivariate	
	OR (95% CI)	*P*-value	OR (95% CI)	*P*-value
Female	0.919 (0.614–1.375)	0.680	0.971 (0.645–1.463)	0.889
HbA1c	1.011 (0.894–1.143)	0.864	1.000 (0.885–1.131)	0.994
Degree of Stenosis	1.383 (0.474–4.039)	0.553	0.392 (0.115–1.338)	0.135
Minimum cross-sectional area	0.919 (0.843–1.002)	0.056	0.948 (0.861–1.044)	0.281
Lesion Length	1.005 (0.992–1.017)	0.472	0.994 (0.977–1.010)	0.455
Calcium volume	1.003 (0.998–1.008)	0.215	1.078 (0.811–1.433)	0.604
CACS (AJ130)	1.000 (1.000–1.000)	0.219	0.998 (0.992–1.005)	0.618
CT-FFR value	0.391 (0.257–0.594)	< 0.001	0.372 (0.233–0.591)	< 0.001

OR, Odds Ratio; CI, confidence interval; CACS, coronary artery calcification score; CT-FFR, computed tomography-derived fractional flow reserve

**Fig. 6:**
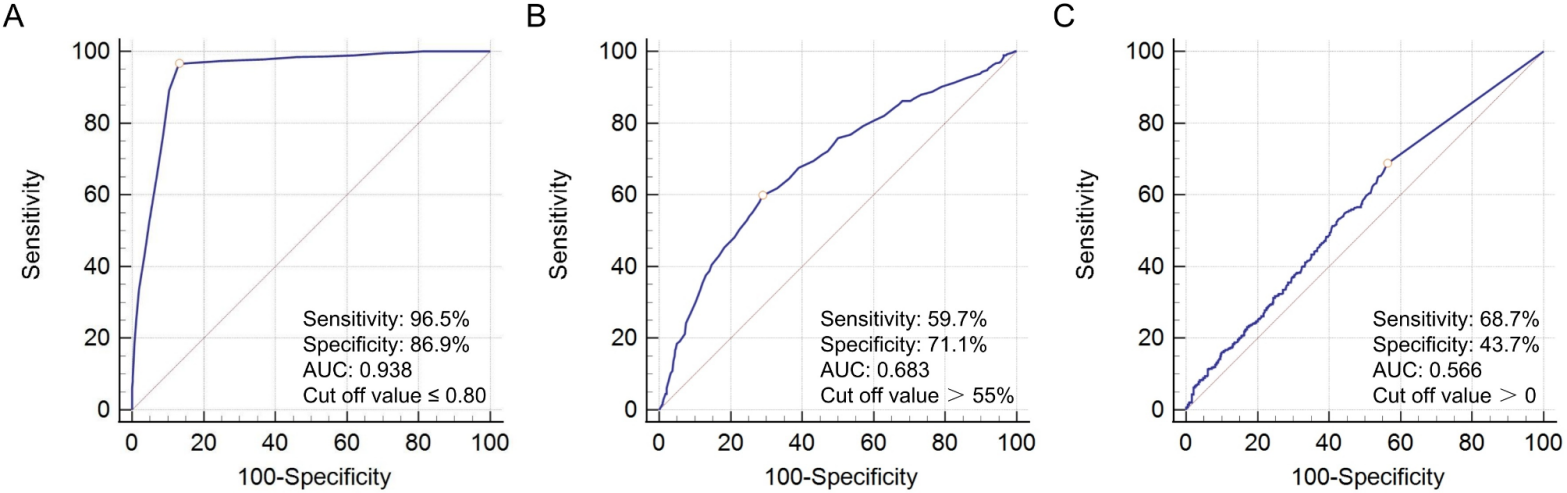
The sensitivity and specificity in predicting MACCE with different indicators. (**A**) CT-FFR; (**B**) The degree of stenosis assessed by CTA; (**C**) CACS. CT-FFR, computed tomography-derived computed fractional flow reserve; CTA, coronary computed tomography angiography; CACS, coronary artery calcification score. CT-FFR (**A**) demonstrates superior diagnostic accuracy compared to the degree of stenosis (**B**) and CACS (**C**) in predicting MACCE

Figure 6 displays the sensitivity and specificity of various indicators in predicting MACCE. For CT-FFR, sensitivity was 96.5%, specificity was 86.9%, and AUC was 0.938. For the degree of stenosis, sensitivity was 59.7%, specificity was 71.1%, and the AUC was 0.683. For CACS, sensitivity was 68.7%, specificity was 43.7%, and the AUC was 0.566. CT-FFR demonstrates superior diagnostic accuracy compared to the degree of stenosis and CACS in predicting MACCE.

## Discussion

In this study, we aimed to evaluate the diagnostic performance of CT-FFR for calcific lesions in patients with T2DM and UA. The key findings are as follows: i) adding CT-FFR to coronary CTA improves diagnostic accuracy due to its superior specificity; ii) CT-FFR has significant predictive value for assessing the prognosis not only in calcified lesions but also in lesions with a CACS score of zero for patients with T2DM and UA.

As coronary calcification is prevalent among diabetic patients, particularly those with T2DM, it is crucial to find a more effective method for evaluating these calcified lesions. Therefore, our study aims to utilize CT-FFR as a complementary approach, integrating both anatomical and functional assessments. The relationship between T2DM and coronary calcific lesions is significant and multifaceted. T2DM is associated with chronic inflammation, oxidative stress, and dyslipidemia, all of which contribute to the development and progression of atherosclerosis [24]. Hyperglycemia leads to the formation of advanced glycation end-products (AGEs), promoting endothelial dysfunction and vascular inflammation [25]. Additionally, insulin resistance affects calcium metabolism, increasing vascular smooth muscle cell proliferation and enhancing calcification within the arterial walls [26]. As a result, patients with T2DM are at a higher risk for calcific lesions, which can further exacerbate cardiovascular complications and lead to adverse outcomes [27]. Understanding these underlying mechanisms is crucial for developing targeted interventions to mitigate cardiovascular risks in this population.

The calcific lesion typically occurs alongside the progression of advanced atherosclerosis. The presence and severity of calcific lesions offer clear evidence of the existence and extent of CAD, serving as a strong predictor of future cardiovascular events. This predictive capability is independent of other cardiovascular risk factors and surpasses that of any other noninvasive biomarker for this condition [28]. However, the connection between calcific lesions and a plaque's likelihood of triggering a cardiovascular event is not yet fully understood. While certain studies have identified microcalcification and spotty calcification as markers of vulnerable plaques [29, 30], others propose that as calcific lesions become more extensive and larger, it reflects a more advanced stage of atherosclerosis, with sheet calcification potentially reducing the risk of plaque rupture [31]. This underscores the need for a combined approach that integrates both anatomical and functional assessments of coronary calcification.

CT-FFR utilizes computer algorithms based on fluid dynamics to calculate FFR from coronary CTA data [32]. The analysis is performed using a standard coronary CTA dataset, with sublingual nitroglycerin administered as per usual protocol. The dataset is then processed, currently requiring a supercomputer for the analysis. Several studies have shown comparable results between the invasive FFR method and the noninvasive coronary CTA-based CT-FFR, while minimizing the impact of imaging artifacts [33, 34]. In our study, CT-FFR values showed a weak correlation with CACS (r = 0.12), indicating that the severity of calcification in patients with T2DM and UA does not accurately identify lesion-specific ischemia. This suggests that relying solely on CTA to assess disease severity and predict prognosis for calcific lesions in patients with T2DM and UA may be inaccurate. Integrating functional assessment alongside anatomical evaluation appears necessary for these patients. Additionally, all patients in our study underwent invasive FFR measurement, and all CT-FFR values were cross-validated against invasive FFR values. The results demonstrated that CT-FFR maintains high diagnostic accuracy at both the patient and vessel levels, even in the presence of calcified lesions. Notably, 40.3% of the enrolled patients had a CACS of 0 (Table 2), and despite the absence of definite calcification, CT-FFR still demonstrated high diagnostic performance in these patients with T2DM. This underscores the broad applicability of CT-FFR in T2DM patients, providing both anatomical and functional insights, which are crucial for guiding clinical decision-making in managing CAD in this population.

In our study, compared to the CT-FFR > 0.8 group, the CT-FFR ≤ 0.8 group has a higher risk of MACCE during a 3-year follow-up period. Meanwhile, there is no statistically significant difference in 3-year MACCE outcomes between patients with both CT-FFR and invasive FFR ≤ 0.8, indicating that CT-FFR does not have an additional impact on prognosis compared to invasive FFR. The study data show that the MACCE incidence in the CT-FFR ≤ 0.8 group is significantly higher than in the CT-FFR > 0.8 group, indicating that CT-FFR ≤ 0.8 can be an effective predictor of myocardial ischemia-related adverse events [35].

For patients with T2DM and UA, CT-FFR values can accurately reflect the degree of coronary stenosis and functional status. The study compared the predictive performance of stenosis degree, CACS, and other indicators with CT-FFR and found that CT-FFR's predictive accuracy is significantly superior to other indicators. This indicates that relying solely on anatomical metrics is inadequate for accurately assessing the functional significance of calcified lesions in patients with T2DM and UA. Therefore, integrating functional assessments such as CT-FFR may be essential. The study findings suggest that a CT-FFR value of ≤ 0.8 serves as an independent risk factor for poor prognosis in patients with T2DM and UA. Given the prognostic importance of CT-FFR, this metric offers valuable insights for treatment decisions in these patients and aids in optimizing clinical management.

### Limitations

Some limitations in this study need to be pointed out. First, due to the nature of retrospective analysis, there is potential for selection bias as the data has already been collected, and researchers cannot control for variables not originally recorded. Second, our study employs a clear cutoff value of 0.8. However, there is a gray zone of 0.75–0.8 that should be considered when using any non-hyperemic FFR measures. By not accounting for this gray zone, our study may oversimplify the interpretation of FFR values and potentially overlook nuanced clinical scenarios. Future research should aim to better integrate this range into the diagnostic framework to enhance the accuracy and reliability of non-hyperemic FFR assessments. Finally, while CT-FFR is validated for use in calcified lesions, its performance in more complex cases or in patients with a combination of calcified and non-calcified lesions might still be a limitation. Further research is needed to evaluate CT-FFR’s effectiveness in such mixed lesion scenarios and to explore any potential limitations in its predictive accuracy in these contexts.

## Conclusion

CT-FFR demonstrated significant diagnostic performance using invasive FFR as the reference standard and proved to be an important predictive tool for assessing prognosis not only in calcified lesions but also in lesions with a CACS score of zero in patients with T2DM and UA. CT-FFR may serve as a valuable tool for guiding treatment decisions in these patients.

## Data Availability

People can get a copy of raw data by emailing the corresponding author.
